# Anti-carbamylated protein antibodies as a new biomarker of erosive joint damage in systemic lupus erythematosus

**DOI:** 10.1186/s13075-018-1622-z

**Published:** 2018-06-14

**Authors:** Fulvia Ceccarelli, Carlo Perricone, Tania Colasanti, Laura Massaro, Enrica Cipriano, Monica Pendolino, Francesco Natalucci, Riccardo Mancini, Francesca Romana Spinelli, Guido Valesini, Fabrizio Conti, Cristiano Alessandri

**Affiliations:** Lupus Clinic, Reumatologia, Dipartimento di Medicina Interna e Specialità Mediche, Sapienza Università di Roma, Viale del Policlinico 155, 00161 Rome, Italy

**Keywords:** Systemic lupus erythematosus, Erosive arthritis, Anti-CarP

## Abstract

**Background:**

The application of more sensitive imaging techniques, such as ultrasonography (US), changed the concept of non-erosive arthritis in systemic lupus erythematosus (SLE), underlining the need for biomarkers to identify patients developing the erosive phenotype. Anti-citrullinated peptide antibodies (ACPA), associated with erosions in inflammatory arthritis, have been identified in about 50% of patients with SLE with erosive arthritis. More recently, anti-carbamylated proteins antibodies (anti-CarP) have been associated with erosive damage in rheumatoid arthritis. We aimed to assess the association between anti-CarP and erosive damage in a large SLE cohort with joint involvement.

**Methods:**

We evaluated 152 patients (male/female patients 11/141; median age 46 years, IQR 16; median disease duration 108 months, IQR 168). All patients underwent blood draw to detect rheumatoid factor (RF) and ACPA (commercial enzyme-linked immunosorbent assay (ELISA) kit), and anti-CarP (“home-made” ELISA, cutoff 340 aU/mL). The bone surfaces of the metacarpophalangeal and proximal interphalangeal joints were assessed by US: the presence of erosions was registered as a dichotomous value (0/1), obtaining a total score (0–20).

**Results:**

The prevalence of anti-CarP was 28.3%, similar to RF (27.6%) and significantly higher than ACPA (11.2%, *p* = 0.003). Erosive arthritis was identified in 25.6% of patients: this phenotype was significantly associated with anti-CarP (*p* = 0.004). Significant correlation between anti-CarP titer and US erosive score was observed (*r* = 0.2, *p* = 0.01).

**Conclusions:**

Significant association was identified between anti-CarP and erosive damage in SLE-related arthritis, in terms of frequency and severity, suggesting that these antibodies can represent a biomarker of severity in patients with SLE with joint involvement.

## Background

Joint involvement is one of the most common manifestations in patients with systemic lupus erythematosus (SLE), with a prevalence ranging from 69 to 95%. This feature significantly influences the patients’ quality of life, possibly leading to disability and impaired functional performance in daily activities [1]. SLE articular involvement could range from arthralgia to severe arthropathy, with inflammation and deformities [1–3].

Novel insights suggest that erosive damage, as assessed by musculoskeletal ultrasonography (US), occurs in up to 47% of patients with SLE [1, 4–8]. It is likely that such an erosive phenotype of SLE arthritis may underlie specific pathogenic mechanisms [1]. Moreover, identification of prognostic biomarkers, able to identify patients at risk of developing this more aggressive phenotype, is mandatory. Autoantibodies associated with inflammatory arthritis, such as rheumatoid arthritis (RA), have also been shown to play a role in SLE arthritis. For instance, anti-citrullinated peptide antibodies (ACPA), which are associated with a worse outcome and a more erosive disease course in RA can be identified in 4.4–27.3% of patients with SLE [9, 10], and their prevalence is even higher in patients with x-ray-detected erosive arthritis, reaching 50% [11, 12].

More recently, anti-carbamylated proteins antibodies (anti-CarP) have been identified in RA, with a prevalence of 16–45% and a significant association with erosive damage and radiographic progression [13–16]. Carbamylation is a non-enzymatic process consisting of the addition of a cyanate group on self-proteins determining a modification in the tertiary structure. This change can cause the generation of new epitopes and the consequent production of autoantibodies [17].

To date, only a few studies have analyzed the prevalence of anti-CarP in patients with SLE with joint involvement [18–20]. Thus, in the present cross-sectional study, we aimed at assessing the association between anti-CarP and US-detected erosive damage in a large cohort of patients with SLE with joint involvement.

## Methods

In the present cross-sectional analysis, we enrolled consecutive patients with SLE with a clinical history of joint involvement (arthralgia or arthritis), attending the Lupus Clinic of the Rheumatology Unit, Sapienza University of Rome (“Sapienza Lupus Cohort”). SLE diagnosis was performed according to the revised 1997 American College of Rheumatology criteria [21]. The study was performed according to the protocol and good clinical practice principles and Declaration of Helsinki statements and was approved by the Ethic committee of the Sapienza University of Rome, Policlinico Umberto I, Rome, Italy. All the patients gave signed informed consent.

The clinical and laboratory data of enrolled patients were collected in a standardized computerized electronically filled form, including demographics, past medical history with the date of diagnosis, co-morbidities and previous and concomitant treatments. We divided patients according to the presence of arthralgia or arthritis. Arthralgia was defined as the presence of recurrent (≥ 3 episodes) or persistent (≥ 6 weeks) pain or stiffness (lasting at least 30 min of at least one joint) in the patient’s clinical history. Arthritis was defined as the occurrence of at least one episode of clinical synovitis (swelling, effusion or tenderness) and at least 30 min of morning stiffness of at least one joint.

The activity of joint involvement was assessed with the swollen-to-tender ratio (STR), previously applied in a SLE cohort [22]. The SLE Disease Activity Index 2000 (SLEDAI-2 k) was used to assess disease activity, while chronic damage was evaluated by the Systemic Lupus International Collaborating Clinics (SLICC) Damage Index (SDI) [23, 24].

### Laboratory evaluation

Each subject underwent peripheral blood sample collection. The study protocol included the determination of autoantibodies and the evaluation of C3 and C4 serum levels. Specifically, anti-nuclear antibodies (ANA) have been determined by means of indirect immunofluorescence (IIF) on HEp-2 (titer ≥ 1:160 or ++, on a scale from + to ++++), anti-double stranded DNA (anti-dsDNA) with IIF on *Crithidia luciliae* (titer ≥ 1:10), extractable nuclear antigen (ENA) (including anti-Ro/SSA, anti-La/SSB, anti-Sm, and anti-RNP) by ELISA, considering titers above the cutoff of the reference laboratory, anti-cardiolipin (anti-CL) (IgG/IgM isotype) by ELISA, in serum or plasma, at medium or high titers (e.g., > 40 GPL or MPL or above the 99th percentile), anti-β2 glycoprotein-I (anti-β2GPI) (IgG/IgM isotype) by ELISA, in serum (above the 99th percentile), and lupus anticoagulant (LA), according to the guidelines of the International Society on Thrombosis and Hemostasis. C3 and C4 serum concentrations were determined by means of radial immunodiffusion.

Moreover, the presence of rheumatoid factor (RF), ACPA, and anti-CarP antibodies was investigated. All autoantibody assays were carried out in triplicate, commercial ELISA kits were used and the results were evaluated according to the manufacturers’ instructions. ACPA titers were obtained using a commercial ELISA kit (DELTA BIOLOGICALS, Rome, Italy). Values above 25 U/mL were considered positive. A solid-phase ELISA kit was used in order to determine RF (Diamedix, Miami, USA). Diluted samples are incubated with purified RF antigen (human IgG) bound to the microtiter well. Any RF-IgM antibody present in the sample binds to the immobilized human IgG to form antigen-antibody complexes. Unbound antibody is washed from the wells and horseradish peroxidase-conjugated anti-human IgM is added. Values above 10 U/mL were considered positive. Anti-CarP antibodies were detected by a “home-made” ELISA, using carbamylated fetal calf serum (Ca-FCS) and non-modified FCS as antigens. Ca-FCS was obtained using the method described by Shi et al. [25]. In brief, Nunc Maxisorp polystyrene plates (Thermo Scientific, Waltham, MA, USA) were coated overnight at 4 °C with non-modified FCS and Ca-FCS (12 μg/ml in 0.05 M Na_2_CO_3_/NaHCO_3_ buffer, pH 9.6). After washing, plates were blocked with phosphate-buffered saline (PBS), containing 1% bovine serum albumin (BSA) (Sigma, Saint Louis, Missouri, USA) for 6 h at 4 °C. Subsequently, the wells were incubated with patients’ serum diluted 1:50 in PBS/0.05% Tween20 (PBS-T)/BSA 1% overnight at 4 °C. Alkaline phosphatase-conjugated anti-human IgG antibodies (Sigma, Saint Louis, MO, USA) were diluted in PBS-T/BSA 1% (1:1000), and incubated for 2 h at room temperature. Paranitrophenyl-phosphate was used as a substrate and the optical density (OD) was measured at 405 nm wavelength. All assays were performed in triplicate and the absorbance of control wells (non-modified FCS) was subtracted to account for non-specific binding. A titration curve of two positive serum samples with medium/high immunoreactivity for Ca-FCS was performed, to assess the performance of the tests and to transform the absorbance of Ca-FCS in arbitrary units per milliliter (aU/mL). The cutoff was established as the mean OD + 3 standard deviations (SD) of 56 age-matched and sex-matched healthy subjects (blood donors) and then the obtained value was converted into arbitrary units per milliliter (corresponding to 340 aU/mL).

#### Ultrasonographic assessment

US imaging was performed in all patients with SLE using a MyLab70 XVG machine (Esaote S.p.A., Florence, Italy) equipped with a 6–18 MHz multifrequency linear array transducer. By using a fixed 18 MHz frequency, the bone surfaces of the metacarpophalangeal (MCP) and proximal interphalangeal (PIP) joints were studied on multiplanar scans according to the European League Against Rheumatism (EULAR) US guidelines [26]. The first through the fifth MCP joints of both hands and the first interphalangeal and the second through the fifth PIP joints of both hands were evaluated. Each joint was scanned in both the longitudinal and transverse planes from the medial to lateral sides on both volar and dorsal aspects, to enable maximum coverage of the joint surface area. The US examination was obtained independently on the same day by two rheumatologists (FC and CP) trained and experienced in musculoskeletal US. Both sonographers were blinded to the US findings of the other observer.

At each joint, according to the Outcome Measures in Rheumatology (OMERACT) definition, the presence of erosions was registered with a dichotomous value (0/1), allowing the possibility to obtain a total score, ranging from 0 to 20 [27]. Moreover, we evaluated the presence of US inflammatory features (synovial effusion and hypertrophy, power Doppler), each measured with a semiquantitative score (0–3), resulting in a total US inflammatory score (0–180).

### Statistical analysis

The statistical analyses were performed using version 5.0 of the GraphPad statistical package. Normally distributed variables were summarized using the mean ± standard deviation (SD), and non-normally distributed variables by the median and interquartile range (IQR). Frequencies were expressed by percentage. Wilcoxon’s matched pairs test and the paired *t* test were performed accordingly. Univariate comparisons between nominal variables were calculated using the chi-square test or Fisher’s exact test, where appropriate. Kruskal-Wallis one-way analysis of variance was applied to evaluate the comparisons between multiple groups. Spearman’s test was used to test correlation. Two-tailed *P* values were reported; *P* values less than 0.05 were considered significant.

Multivariate analysis was performed using binary logistic regression. The results are presented as standard error (SE) with the 95% confidence interval (CI). In order to perform the multivariate analysis, we used a step-forward model including, progressively, those variables with *P* < 0.1 (to also include those with a trend toward an association) to have a stronger model. Area under the receiver operating characteristic curve (ROC-AUC) was analyzed to evaluate the sensitivity and specificity of anti-CarP and ACPA in identifying the presence of erosive arthritis. Interobserver reproducibility was determined using κ statistics, and κ values were evaluated according to the method of Landis and Koch [28].

## Results

We evaluated 152 patients with SLE with joint involvement (male/female patients 11/141; median age 46 years, IQR 16; median disease duration 108 months, IQR 168). Demographic, clinical and immunological features of enrolled patients, according to disease course, are reported in Table 1.

**Table 1:** Demographic features, clinical and laboratory manifestations and treatments in152 patients with SLE

	Value
Clinical manifestations – *N* (%)	
Skin involvement	115 (75.7)
Serositis	31 (20.4)
Hematological manifestations	99 (65.1)
Neuropsychiatric involvement	15 (9.9)
Renal involvement	33 (21.7)
Laboratory manifestations – *N* (%)	
Anti-dsDNA	114 (78.9)
Anti-Sm	28 (18.4)
Anti-SSA	62 (40.8)
Anti-SSB	35 (23.0)
Anti-RNP	24 (15.8)
Anti-cardiolipin IgG/IgM	44 (28.9)
Anti-β2GPI IgG/IgM	38 (25.0)
Lupus anticoagulant	39 (25.6)
Low C3/C4 levels	81 (53.3)
Treatments – *N* (%)	
Glucocorticoids	140 (92.1)
Hydroxychloroquine	144 (94.7)
Cyclosporine A	34 (22.4)
Methotrexate	59 (38.8)
Cyclophosphamide	6 (3.9)
Mycophenolate mofetil	43 (28.3)
Azathioprine	29 (19.1)
Rituximab	4 (2.6)
Belimumab	9 (5.9)

*SLE* systemic lupus erythematosus

Skin involvement represented the most frequent SLE-related manifestation identified in 75.8% of patients, and more than 90% of patients were treated by glucocorticoids and hydroxychloroquine. During the disease course, 47 patients (30.9%) had arthralgia. The remaining 105 patients experienced at least one episode of arthritis, and Jaccoud’s arthropathy was described in 17 patients (16.2%). The mean ± SD interval between the arthritis onset and the blood sample collection was 14.5 ± 10.3 years.

**Fig. 1:**
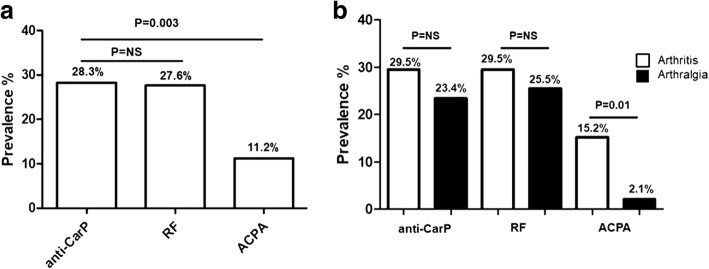
**a** Prevalence of anti-carbamylated proteins antibodies (anti-CarP), rheumatoid factor (RF) and anti-citrullinated peptide antibodies (ACPA) in the enrolled patients with systemic lupus erythematosus (SLE) (*N* = 152). **b** Prevalence of anti-CarP, RF and ACPA according to the phenotype of joint involvement (arthritis/arthralgia). NS, not significant

At the time of the enrollment, the median SLEDAI-2 k was 2 (range 0–10, IQR 4). Concerning the joint involvement activity, the entire population had a STR (mean ± SD) = 0.34 ± 0.5 (median 0.05, IQR 0.7). Figure 1a shows the prevalence of anti-CarP, RF and ACPA in the study cohort.

Anti-CarP-positive (*n* = 42, 28.3%) and RF-positive (*n* = 42, 27.6%) patients were significantly more prevalent than ACPA-positive patients (*n* = 17, 11.2%; *P* = 0.003). The median anti-CarP titer was 185.5 UA/ml (IQR 333.9), the median ACPA was 0 UI/ml (IQR 3.42) and the median RF was 7 (IQR 20.7). In Fig. 1b data are reported on the frequency of anti-CarP, RF and ACPA according to joint involvement phenotype. The prevalence of anti-CarP and RF was similar in patients with SLE with arthritis and arthralgia (anti-CarP, 31 patients with arthritis (29.5%) versus 11 patients (23.4%) with arthralgia, *P* value not significant; RF, 31 patients with arthritis (29.5%) versus 12 patients with arthralgia (25.5%), *P* value not significant). Conversely, ACPA were significantly more frequent in patients with arthritis (16 patients, 15.2%) in comparison with those experiencing arthralgia (1 patient, 2.1%; *P* = 0.01).

**Fig. 2:**
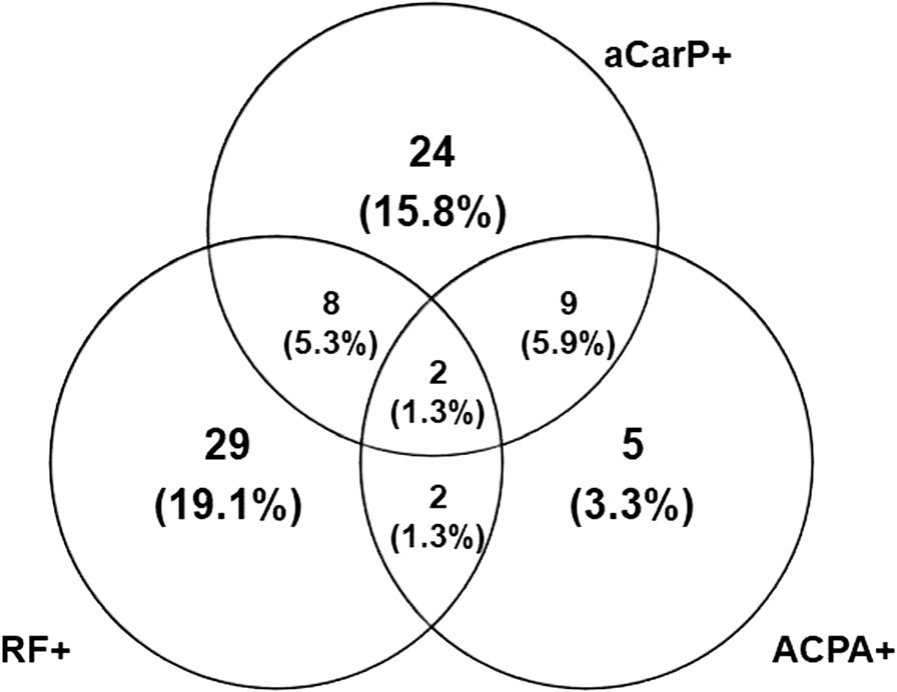
Distribution of single-positive, double-positive and triple-positive patients with systemic lupus erythematosus. RF, rheumatoid factor; ACPA, anti-citrullinated protein antibodies; aCarP, anti-carbamylated protein antibodies

Figure 2 reports the number and the percentage of single-positive, double-positive and triple-positive patients with SLE: 73 patients (48.0%%) were negative for all autoantibodies. Of note, anti-CarP antibodies were identified in 24.5% of ACPA-negative and RF-negative patients.

Anti-CarP-positive patients had significantly higher prevalence of ACPA, compared to anti-CarP-negative patients (23.2% versus 6.4%, *P* = 0.001).

Anti-CarP titers were significantly correlated with C-reactive protein (CRP) values (*r* = 0.3, *P* = 0.02) and STR values (*r* = 0.3, *P* = 0.02). Moreover, also ACPA titers were significantly correlated with STR values (*r* = 0.1, *P* = 0.003).

Interobserver agreement was statistically significant when bone erosions were evaluated (*P* < 0.0001). A comparison of the results from the two sonographers showed that the overall unweighted κ value for the examined joints was 0.72 (agreement in 87.5% of examinations).

**Fig. 3:**
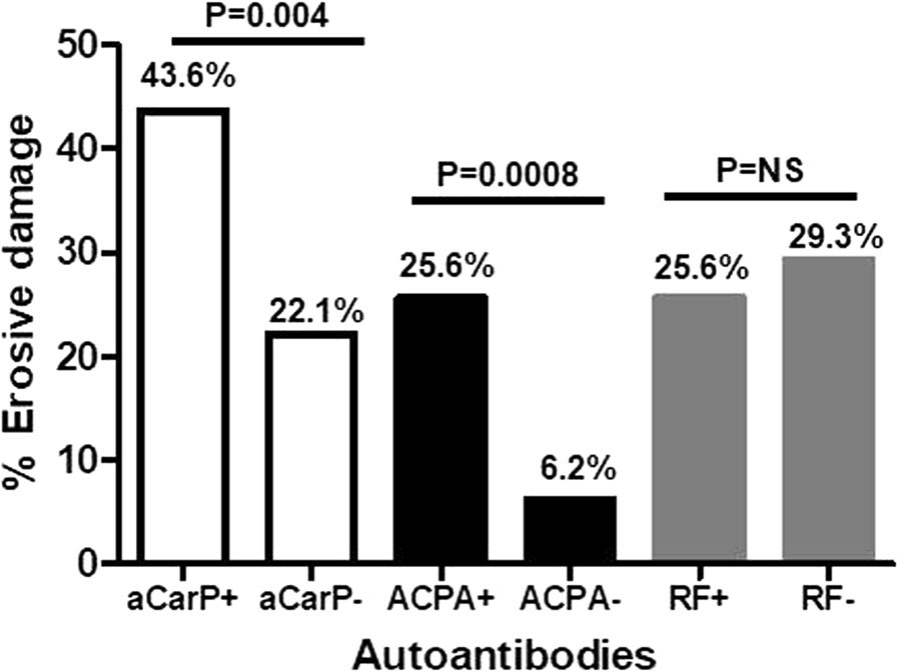
Prevalence of erosive damage according to the autoantibody status. aCarP, anti-carbamylated protein antibodies; ACPA, anti-citrullinated protein antibodies; RF, rheumatoid factor; NS, not significant

US evaluation of the enrolled patients with SLE allowed the identification of erosive arthritis in 39 patients with SLE (25.6%), with a mean ± SD erosive score of 2.9 ± 2.7 (median 2, IQR 3). All the patients with erosive damage reported at least one episode of clinically evident synovitis during the disease course. The prevalence of erosive damage was significantly higher in anti-CarP-positive compared with anti-CarP-negative patients (43.6% versus 22.1%, *P* = 0.004, Fig. 3). Moreover, ACPA-positive patients had significantly higher prevalence of erosive damage than ACPA-negative patients (25.6% versus 6.2%, *P* = 0.0008), while a similar prevalence was observed in RF-positive vs RF-negative patients with SLE(25.6% versus 29.3%, *P* value not significant).

**Fig. 4:**
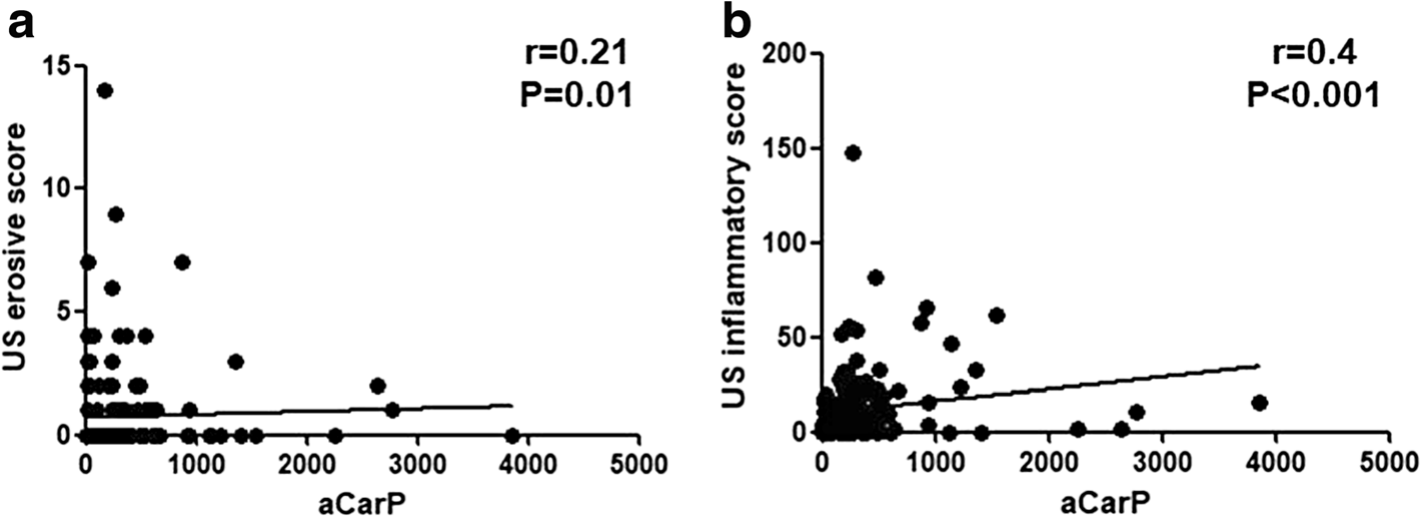
Correlation between anti-carbamylated protein antibodies (anti-CarP) titers and ultrasound (US) erosive (**a**) and inflammatory score (**b**). STR, swollen-to-tender ratio

When considering the subgroup of anti-CarP single-positive patients, erosive damage was identified in 25% of patients. Interestingly, anti-CarP titers were significantly correlated with the US erosive score (*r* = 0.21, *P* = 0.01; Fig. 4a); conversely, there was no association between erosive score and ACPA or RF titers. The median US total inflammatory score was 4 (IQR 14). There was significant correlation between this score and anti-CarP titer (*r* = 0.4, *P* < 0.001; Fig. 4b).

**Fig. 5:**
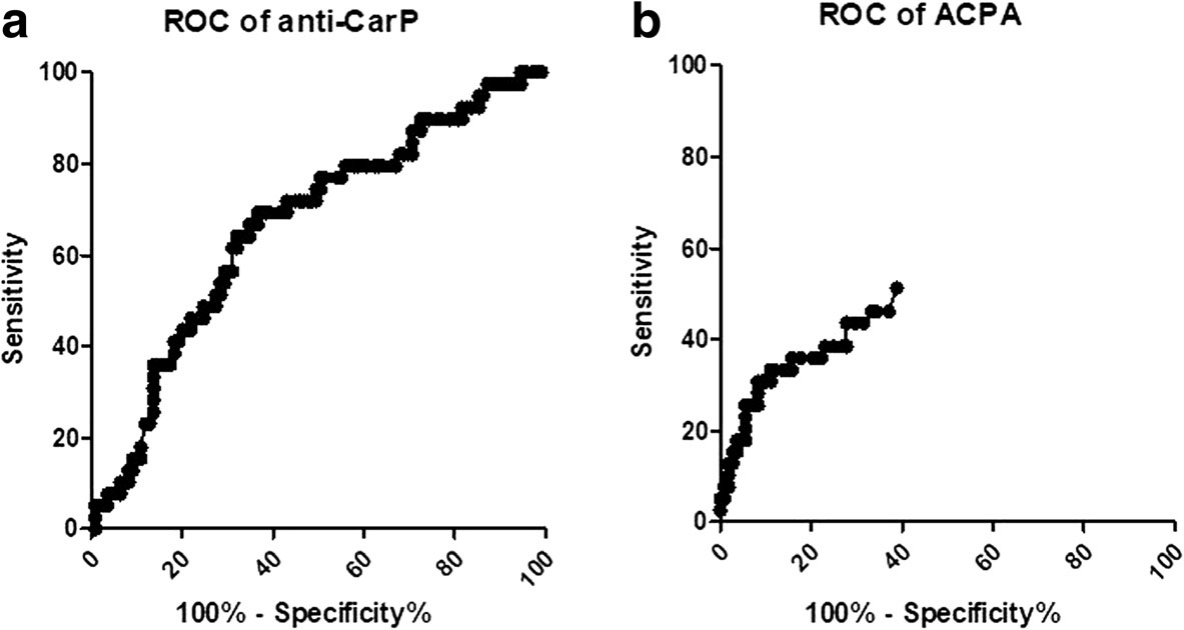
ROC curve analysis for anti-carbamylated protein antibodies (anti-CarP) (**a**) and anti-citrullinated protein antibodies (ACPA) (**b**)

According to the positivity cutoff for anti-CarP to distinguish between patients with and without erosive arthritis (Fig. 5). For anti-CarP, with a cutoff of 354.4 aU/mL the AUC was 0.66 (SE 0.05, sensitivity 43.6%, specificity 79.8%, likelihood ratio 2.16; Fig. 5a). For ACPA, we the AUC was 0.59 (SE 0.05, sensitivity 25.6%, specificity 92.6%, likelihood ratio 3.46; Fig. 5b) for a cutoff value of 23.2 UI/mL. The multivariate analysis confirmed the association between erosive arthritis and anti-CarP (*P* = 0.04, SE 0.49, 95% CI 1.0–6.9) and Jaccoud arthropathy (*P* = 0.01, SE 0.6, 95% CI 1.4–15.5).

## Discussion

The present study is the first specifically designed to evaluate the role of anti-CarP as a biomarker of erosive damage in patients with SLE joint involvement. By evaluating a large single-center cohort, we identified a significant association between anti-CarP and erosive damage. Interestingly, our results suggest that anti-CarP may prove useful as a biomarker of activity, as demonstrated by correlation with the US inflammatory score, CRP and STR.

Despite the relatively high frequency of joint involvement in patients with SLE, the pathogenetic picture remains incomplete and specific biomarkers are lacking [1]. Several studies suggest that a predisposing genetic background is detrimental in the development of joint involvement [29–31]. For instance, Ciccacci et al. observed an association between joint involvement and mir146a rs2910164 gene variant [32]. These genetic modifications could contribute to T cell activation through the interaction with antigen presenting cells via T cell receptor and co-stimulatory molecules. This activation finally leads to B cell proliferation and production of autoantibodies [1]. Similar to RA, proteins deriving from post-translational modifications, such as citrullination or carbamylation, could activate this process [1, 17].

The role of ACPA as biomarkers of a more aggressive disease phenotype has been widely investigated both in RA and SLE. Indeed, these autoantibodies can be frequently found in patients with SLE with x-ray-detected erosive arthritis [11]. Nonetheless, most patients with SLE with erosive arthritis are ACPA-negative, suggesting a different pathogenic scenario [12].

Carbamylation occurs in several inflammatory conditions including RA and psoriatic arthritis [33]. In RA, anti-CarP antibodies have been identified in 16% of seronegative (ACPA and RF negative) patients and seem to correlate with bone erosions and radiographic progression. Anti-CarP have also been evaluated in patients affected with SLE. Specifically, Ziegelasch et al. identified association between anti-CarP and radiographically detected erosions in a small cohort of patients with SLE [19]. We previously identified anti-CarP in 46.1% of patients with SLE with joint involvement, a prevalence matching that in patients with RA and significantly higher than that in healthy controls (2.4%). We also found that anti-CarP-positive patients were significantly more frequent than ACPA-positive patients [20].

With the present study, we add a piece to the puzzle suggesting that anti-CarP may be involved in the development of erosive damage in SLE, since anti-CarP-positive patients are more prevalent among those with US-detected erosions and anti-CarP titers correlate with US erosive score. This result was confirmed in the multivariate analysis. Moreover, the ROC curve analysis demonstrated high specificity of anti-CarP to differentiate between patients with and without erosive arthritis.

The pathogenetic mechanism leading to erosive damage has been investigated, especially in RA. In this context autoantibodies, including ACPA and anti-CarP, seem to play a pathogenetic role by acting on bone-resorbing osteoclasts. ACPA have been shown to bind to osteoclasts and osteoclast precursors, promoting osteoclast differentiation and osteolytic function in vit*ro* [34]. Moreover, binding of ACPA to osteoclast precursors could promote the release of pro-inflammatory and pro-osteoclastogenic cytokines, such as TNF, linking autoimmunity and inflammation [34]. We could hypothesize that such a mechanism occurs in anti-CarP-positive patients with SLE. This scenario seems to be confirmed by the correlation between anti-CarP titers and inflammatory parameters, such as US inflammatory score, CRP and STR, as observed in our cohort.

Paralleling ACPA behavior, anti-CarP could induce the production of pro-inflammatory cytokines, leading to a more active phenotype. Nonetheless, anti-CarP seem specifically involved in joint involvement development rather than being markers of disease activity, since they do not correlate with disease activity indices, such as the SLEDAI-2 k. We may hypothesize that NETosis-derived myeloperoxidase could locally determine a pro-carbamylation milieu, leading to the break of immune tolerance and to the production of antibodies directed against carbamylated proteins [20]. NETosis, the specific neutrophil death, plays a fundamental role in SLE pathogenesis: several data have shown increased NETosis in patients with SLE, which significantly correlated with disease activity [35].

Finally, we have chosen to use US assessment, given its high sensitivity in the detection of bone erosions and soft-tissue lesions. Indeed, US is more sensitive than x-ray in detecting bone erosions at the MCP and metatarsophalangeal joints in patients affected by RA, compared to magnetic resonance imaging and computed tomography, especially during the early disease phase [36, 37].

## Conclusions

The results of the present study suggest that anti-CarP could be considered as a candidate biomarker of severity and activity in SLE joint involvement. Further studies are needed to confirm this issue and to identify the pathogenetic mechanism underlining this association.
